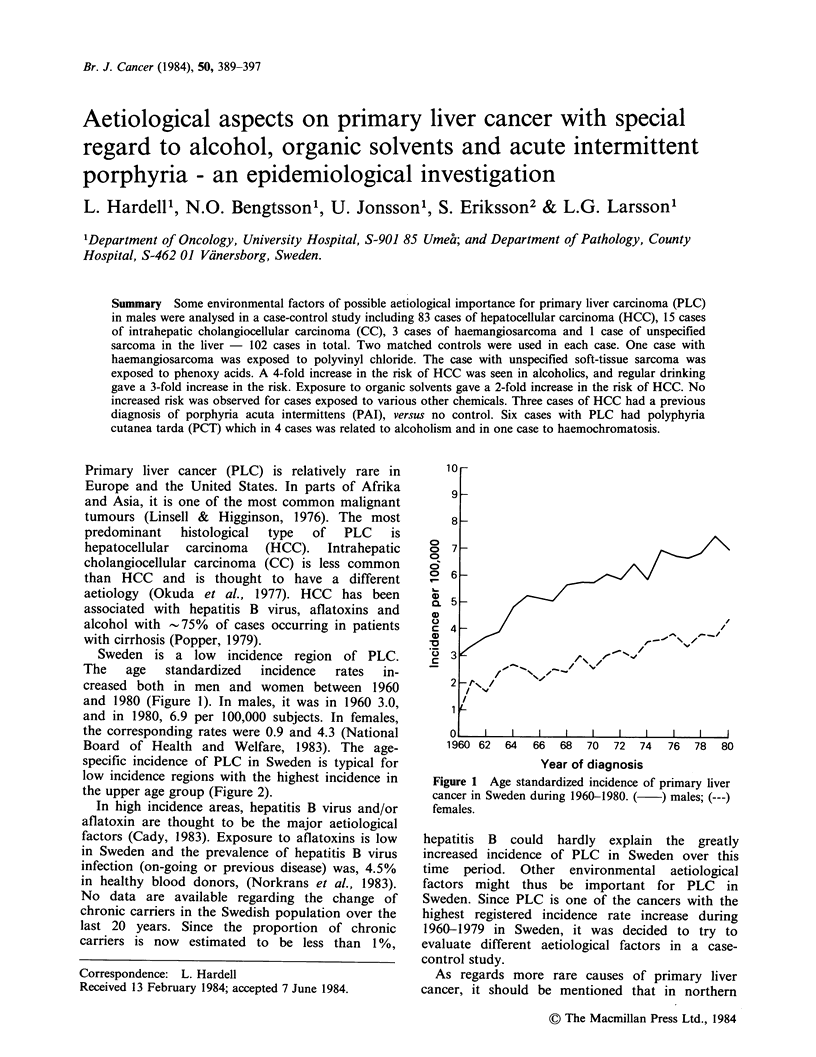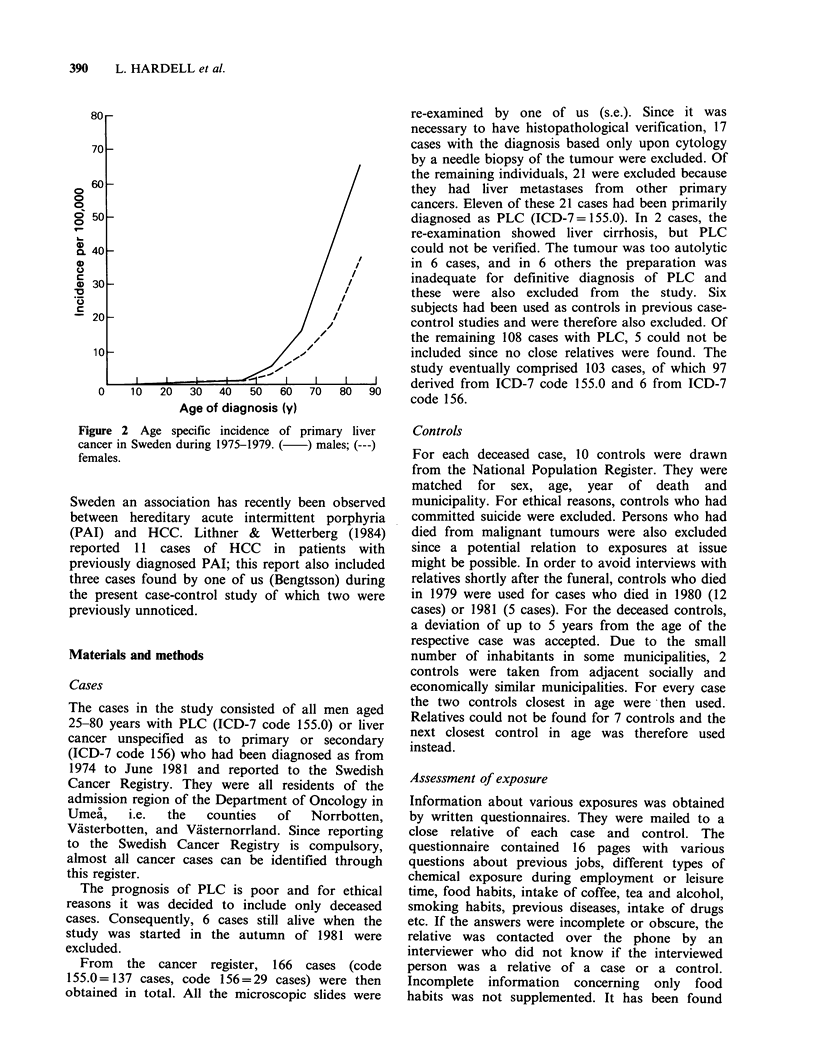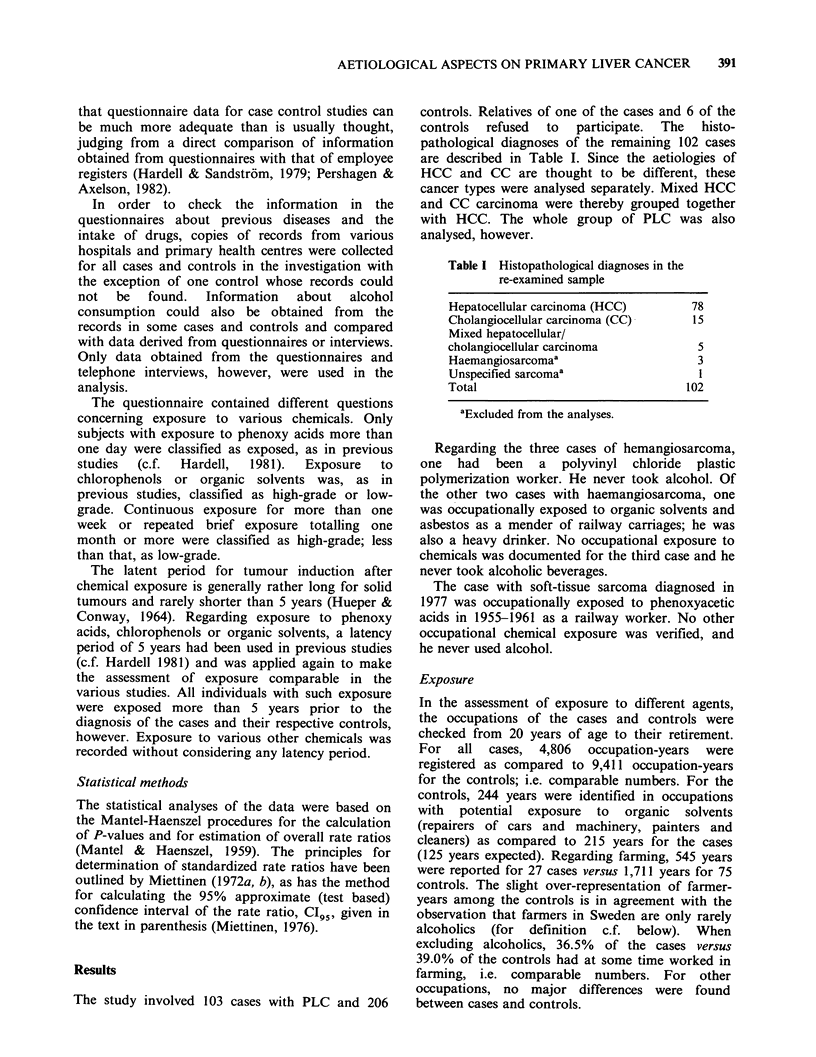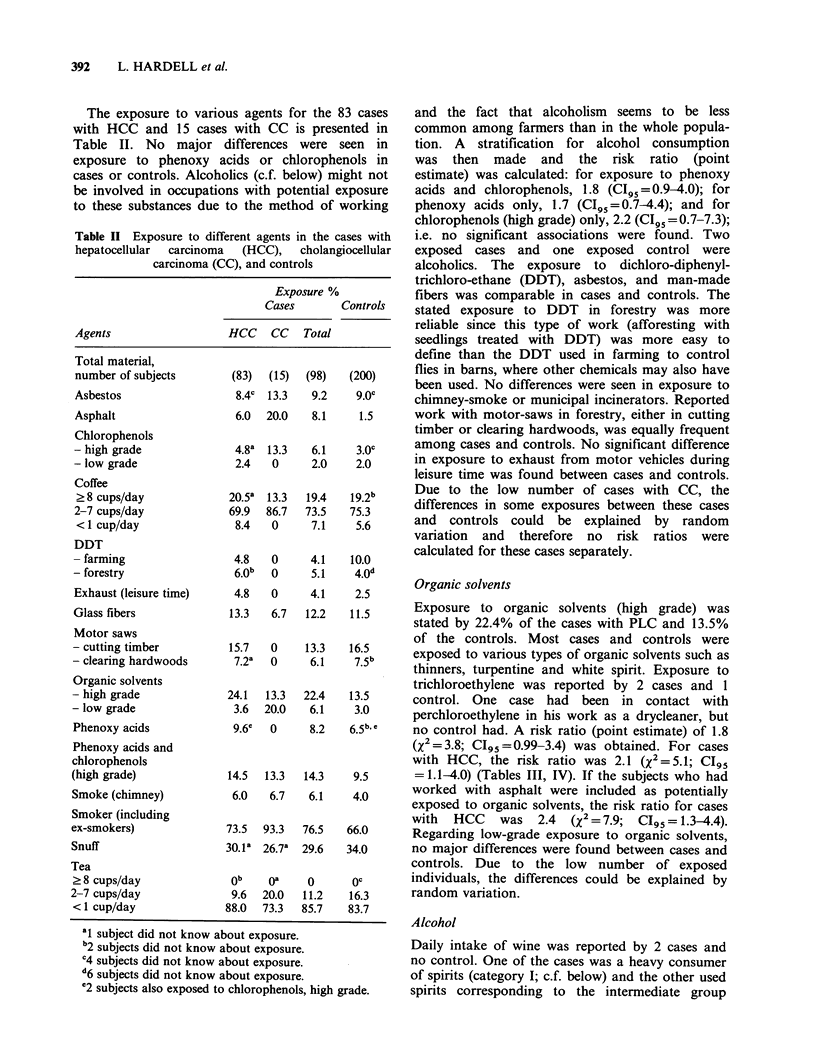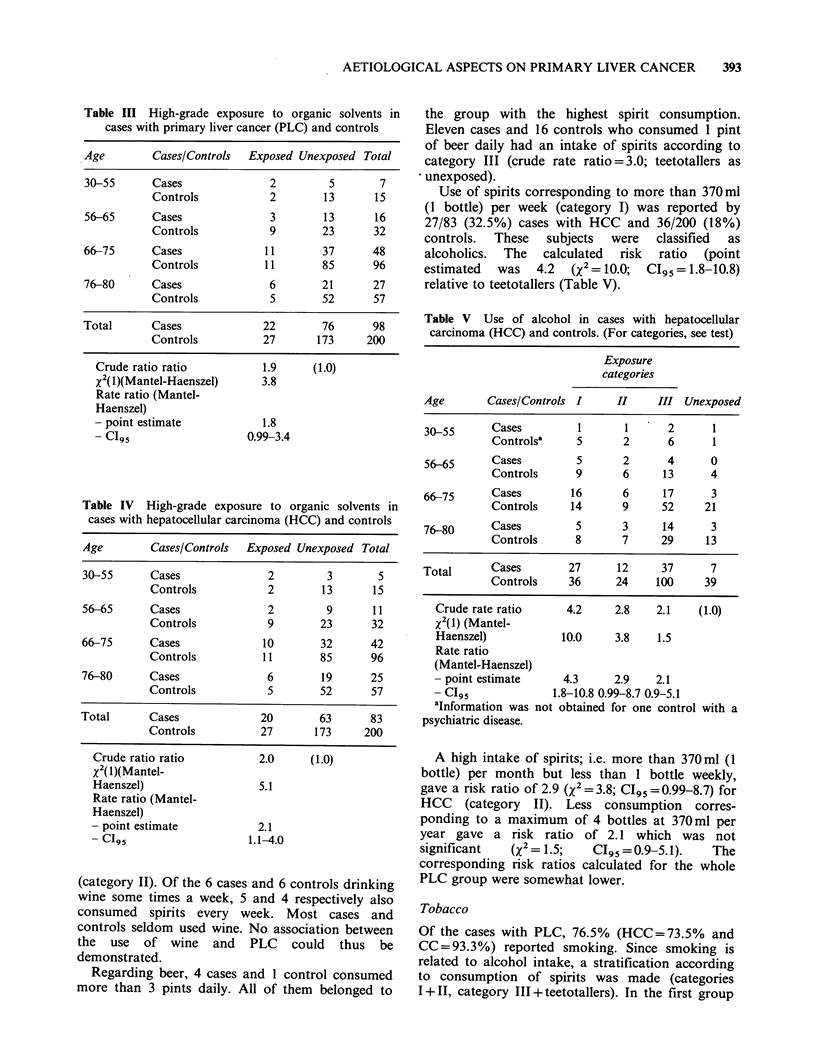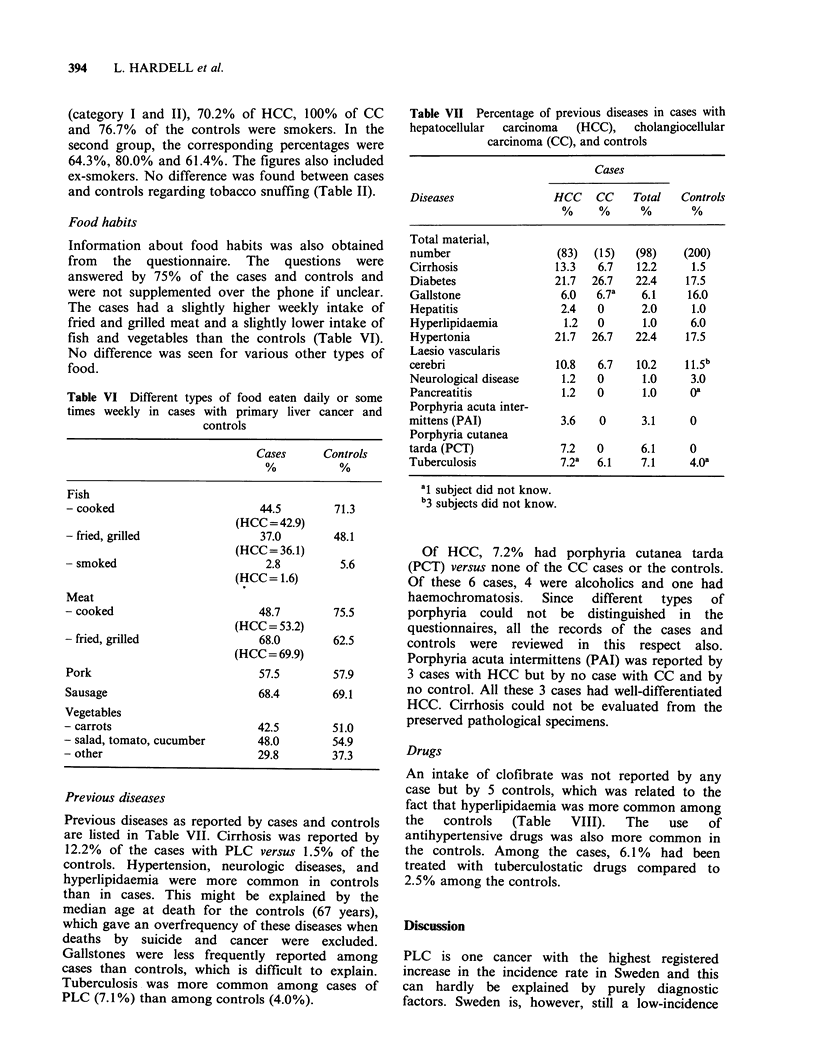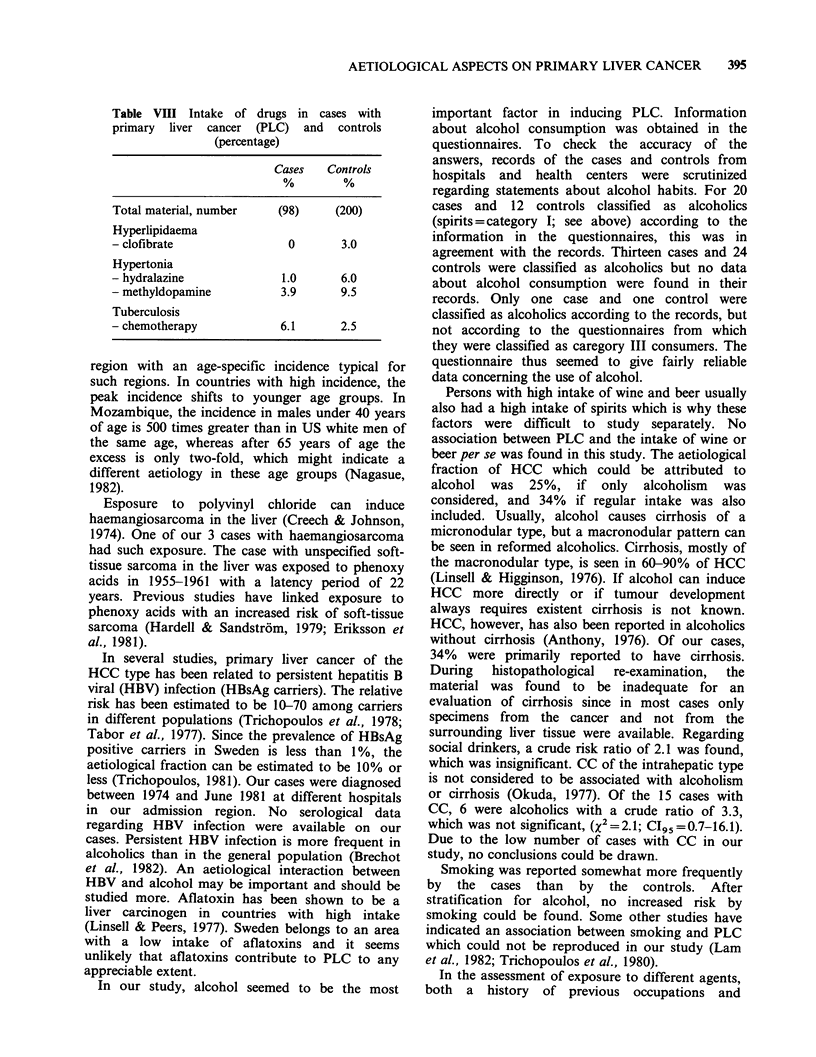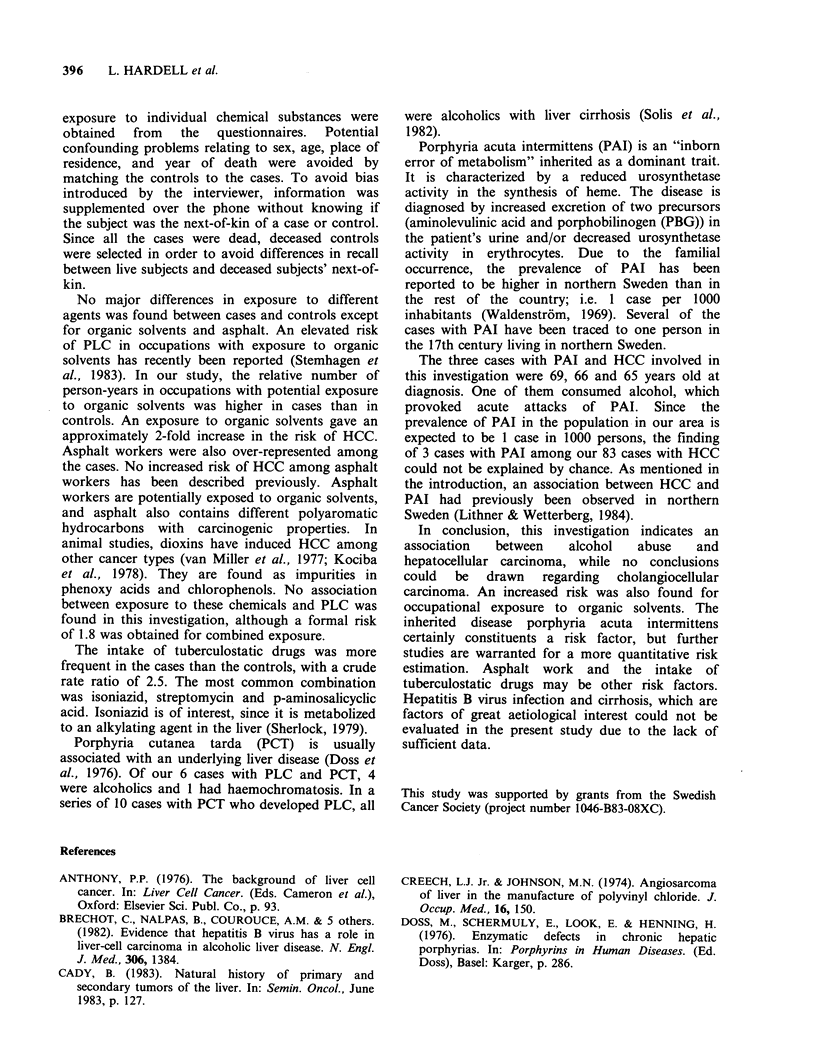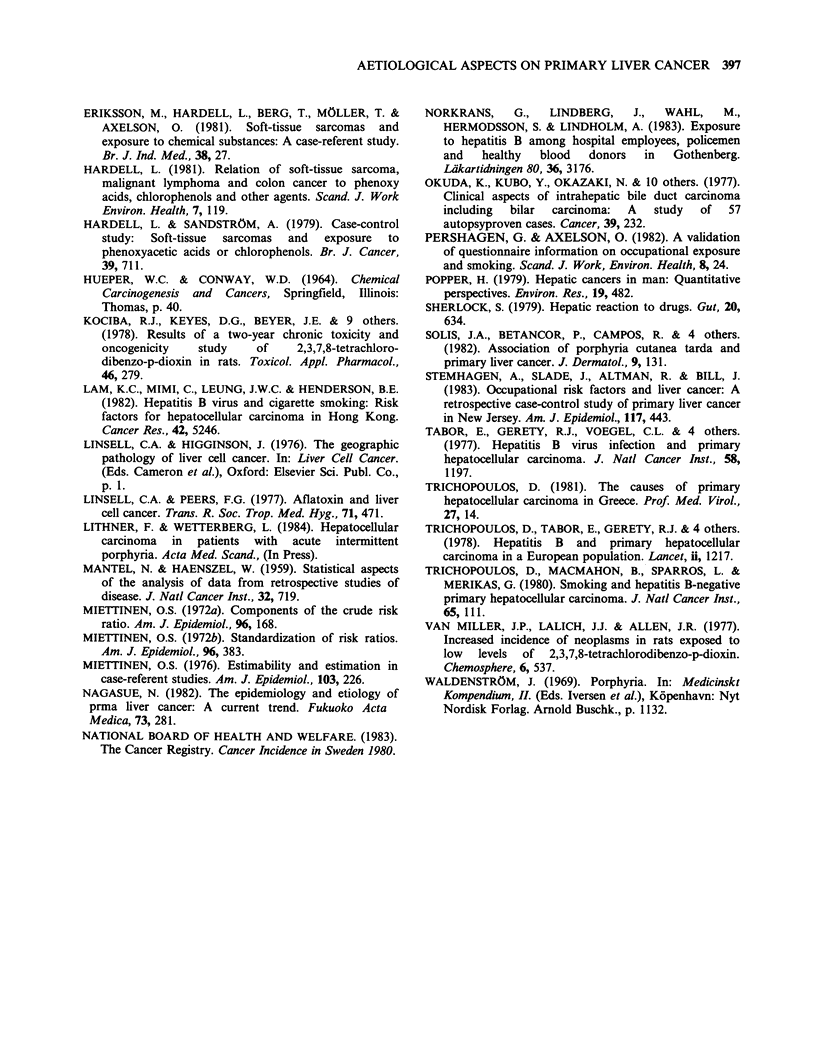# Aetiological aspects on primary liver cancer with special regard to alcohol, organic solvents and acute intermittent porphyria--an epidemiological investigation.

**DOI:** 10.1038/bjc.1984.188

**Published:** 1984-09

**Authors:** L. Hardell, N. O. Bengtsson, U. Jonsson, S. Eriksson, L. G. Larsson

## Abstract

Some environmental factors of possible aetiological importance for primary liver carcinoma (PLC) in males were analysed in a case-control study including 83 cases of hepatocellular carcinoma (HCC), 15 cases of intrahepatic cholangiocellular carcinoma (CC), 3 cases of haemangiosarcoma and 1 case of unspecified sarcoma in the liver--102 cases in total. Two matched controls were used in each case. One case with haemangiosarcoma was exposed to polyvinyl chloride. The case with unspecified soft-tissue sarcoma was exposed to phenoxy acids. A 4-fold increase in the risk of HCC was seen in alcoholics, and regular drinking gave a 3-fold increase in the risk. Exposure to organic solvents gave a 2-fold increase in the risk of HCC. No increased risk was observed for cases exposed to various other chemicals. Three cases of HCC had a previous diagnosis of porphyria acuta intermittens (PAI), versus no control. Six cases of HCC had a previous diagnosis of porphyria acuta intermittens (PAI), versus no control. Six cases with PLC had polyphyria cutanea tarda (PCT) which in 4 cases was related to alcoholism and in one case to haemochromatosis.


*Br. J. Cancer* (1984), **50**, 389–397

### Aetiological aspects on primary liver cancer with special regard to alcohol, organic solvents and acute intermittent porphyria - an epidemiological investigation

L. Hardell[1], N.O. Bengtsson[1], U. Jonsson[1], S. Eriksson[2] & L.G. Larsson[1]

[1]*Department of Oncology, University Hospital, S-901 85 Umeå; and Department of Pathology, County Hospital, S-462 01 Vänersborg, Sweden.*

**Summary** Some environmental factors of possible aetiological importance for primary liver carcinoma (PLC) in males were analysed in a case-control study including 83 cases of hepatocellular carcinoma (HCC), 15 cases of intrahepatic cholangiocellular carcinoma (CC), 3 cases of haemangiosarcoma and 1 case of unspecified sarcoma in the liver — 102 cases in total. Two matched controls were used in each case. One case with haemangiosarcoma was exposed to polyvinyl chloride. The case with unspecified soft-tissue sarcoma was exposed to phenoxy acids. A 4-fold increase in the risk of HCC was seen in alcoholics, and regular drinking gave a 3-fold increase in the risk. Exposure to organic solvents gave a 2-fold increase in the risk of HCC. No increased risk was observed for cases exposed to various other chemicals. Three cases of HCC had a previous diagnosis of porphyria acuta intermittens (PAI), *versus* no control. Six cases with PLC had polyphyria cutanea tarda (PCT) which in 4 cases was related to alcoholism and in one case to haemochromatosis.

Primary liver cancer (PLC) is relatively rare in Europe and the United States. In parts of Afrika and Asia, it is one of the most common malignant tumours (Linsell & Higginson, 1976). The most predominant histological type of PLC is hepatocellular carcinoma (HCC). Intrahepatic cholangiocellular carcinoma (CC) is less common than HCC and is thought to have a different aetiology (Okuda *et al.*, 1977). HCC has been associated with hepatitis B virus, aflatoxins and alcohol with ~75% of cases occurring in patients with cirrhosis (Popper, 1979).

Sweden is a low incidence region of PLC. The age standardized incidence rates increased both in men and women between 1960 and 1980 (Figure 1). In males, it was in 1960 3.0, and in 1980, 6.9 per 100,000 subjects. In females, the corresponding rates were 0.9 and 4.3 (National Board of Health and Welfare, 1983). The age-specific incidence of PLC in Sweden is typical for low incidence regions with the highest incidence in the upper age group (Figure 2).

In high incidence areas, hepatitis B virus and/or aflatoxin are thought to be the major aetiological factors (Cady, 1983). Exposure to aflatoxins is low in Sweden and the prevalence of hepatitis B virus infection (on-going or previous disease) was, 4.5% in healthy blood donors, (Norkrans *et al.*, 1983). No data are available regarding the change of chronic carriers in the Swedish population over the last 20 years. Since the proportion of chronic carriers is now estimated to be less than 1%,

**Figure 1** Age standardized incidence of primary liver cancer in Sweden during 1960–1980. (——) males; (---) females.

hepatitis B could hardly explain the greatly increased incidence of PLC in Sweden over this time period. Other environmental aetiological factors might thus be important for PLC in Sweden. Since PLC is one of the cancers with the highest registered incidence rate increase during 1960–1979 in Sweden, it was decided to try to evaluate different aetiological factors in a case-control study.

As regards more rare causes of primary liver cancer, it should be mentioned that in northern

Correspondence: L. Hardell
Received 13 February 1984; accepted 7 June 1984.

© The Macmillan Press Ltd., 1984

**Figure 2** Age specific incidence of primary liver cancer in Sweden during 1975–1979. (——) males; (---) females.

Sweden an association has recently been observed between hereditary acute intermittent porphyria (PAI) and HCC. Lithner & Wetterberg (1984) reported 11 cases of HCC in patients with previously diagnosed PAI; this report also included three cases found by one of us (Bengtsson) during the present case-control study of which two were previously unnoticed.

### Materials and methods

### *Cases*

The cases in the study consisted of all men aged 25–80 years with PLC (ICD-7 code 155.0) or liver cancer unspecified as to primary or secondary (ICD-7 code 156) who had been diagnosed as from 1974 to June 1981 and reported to the Swedish Cancer Registry. They were all residents of the admission region of the Department of Oncology in Umeå, i.e. the counties of Norrbotten, Västerbotten, and Västernorrland. Since reporting to the Swedish Cancer Registry is compulsory, almost all cancer cases can be identified through this register.

The prognosis of PLC is poor and for ethical reasons it was decided to include only deceased cases. Consequently, 6 cases still alive when the study was started in the autumn of 1981 were excluded.

From the cancer register, 166 cases (code 155.0 = 137 cases, code 156 = 29 cases) were then obtained in total. All the microscopic slides were re-examined by one of us (s.e.). Since it was necessary to have histopathological verification, 17 cases with the diagnosis based only upon cytology by a needle biopsy of the tumour were excluded. Of the remaining individuals, 21 were excluded because they had liver metastases from other primary cancers. Eleven of these 21 cases had been primarily diagnosed as PLC (ICD-7 = 155.0). In 2 cases, the re-examination showed liver cirrhosis, but PLC could not be verified. The tumour was too autolytic in 6 cases, and in 6 others the preparation was inadequate for definitive diagnosis of PLC and these were also excluded from the study. Six subjects had been used as controls in previous case-control studies and were therefore also excluded. Of the remaining 108 cases with PLC, 5 could not be included since no close relatives were found. The study eventually comprised 103 cases, of which 97 derived from ICD-7 code 155.0 and 6 from ICD-7 code 156.

### *Controls*

For each deceased case, 10 controls were drawn from the National Population Register. They were matched for sex, age, year of death and municipality. For ethical reasons, controls who had committed suicide were excluded. Persons who had died from malignant tumours were also excluded since a potential relation to exposures at issue might be possible. In order to avoid interviews with relatives shortly after the funeral, controls who died in 1979 were used for cases who died in 1980 (12 cases) or 1981 (5 cases). For the deceased controls, a deviation of up to 5 years from the age of the respective case was accepted. Due to the small number of inhabitants in some municipalities, 2 controls were taken from adjacent socially and economically similar municipalities. For every case the two controls closest in age were then used. Relatives could not be found for 7 controls and the next closest control in age was therefore used instead.

### *Assessment of exposure*

Information about various exposures was obtained by written questionnaires. They were mailed to a close relative of each case and control. The questionnaire contained 16 pages with various questions about previous jobs, different types of chemical exposure during employment or leisure time, food habits, intake of coffee, tea and alcohol, smoking habits, previous diseases, intake of drugs etc. If the answers were incomplete or obscure, the relative was contacted over the phone by an interviewer who did not know if the interviewed person was a relative of a case or a control. Incomplete information concerning only food habits was not supplemented. It has been found that questionnaire data for case control studies can be much more adequate than is usually thought, judging from a direct comparison of information obtained from questionnaires with that of employee registers (Hardell & Sandström, 1979; Pershagen & Axelson, 1982).

In order to check the information in the questionnaires about previous diseases and the intake of drugs, copies of records from various hospitals and primary health centres were collected for all cases and controls in the investigation with the exception of one control whose records could not be found. Information about alcohol consumption could also be obtained from the records in some cases and controls and compared with data derived from questionnaires or interviews. Only data obtained from the questionnaires and telephone interviews, however, were used in the analysis.

The questionnaire contained different questions concerning exposure to various chemicals. Only subjects with exposure to phenoxy acids more than one day were classified as exposed, as in previous studies (c.f. Hardell, 1981). Exposure to chlorophenols or organic solvents was, as in previous studies, classified as high-grade or low-grade. Continuous exposure for more than one week or repeated brief exposure totalling one month or more were classified as high-grade; less than that, as low-grade.

The latent period for tumour induction after chemical exposure is generally rather long for solid tumours and rarely shorter than 5 years (Hueper & Conway, 1964). Regarding exposure to phenoxy acids, chlorophenols or organic solvents, a latency period of 5 years had been used in previous studies (c.f. Hardell 1981) and was applied again to make the assessment of exposure comparable in the various studies. All individuals with such exposure were exposed more than 5 years prior to the diagnosis of the cases and their respective controls, however. Exposure to various other chemicals was recorded without considering any latency period.

### *Statistical methods*

The statistical analyses of the data were based on the Mantel-Haenszel procedures for the calculation of *P*-values and for estimation of overall rate ratios (Mantel & Haenszel, 1959). The principles for determination of standardized rate ratios have been outlined by Miettinen (1972*a, b*), as has the method for calculating the 95% approximate (test based) confidence interval of the rate ratio, $CI_{95}$, given in the text in parenthesis (Miettinen, 1976).

### Results

The study involved 103 cases with PLC and 206 controls. Relatives of one of the cases and 6 of the controls refused to participate. The histopathological diagnoses of the remaining 102 cases are described in Table I. Since the aetiologies of HCC and CC are thought to be different, these cancer types were analysed separately. Mixed HCC and CC carcinoma were thereby grouped together with HCC. The whole group of PLC was also analysed, however.

**Table I** Histopathological diagnoses in the re-examined sample

| | |
|---|---|
| Hepatocellular carcinoma (HCC) | 78 |
| Cholangiocellular carcinoma (CC) | 15 |
| Mixed hepatocellular/ cholangiocellular carcinoma | 5 |
| Haemangiosarcoma[a] | 3 |
| Unspecified sarcoma[a] | 1 |
| Total | 102 |

[a]Excluded from the analyses.

Regarding the three cases of hemangiosarcoma, one had been a polyvinyl chloride plastic polymerization worker. He never took alcohol. Of the other two cases with haemangiosarcoma, one was occupationally exposed to organic solvents and asbestos as a mender of railway carriages; he was also a heavy drinker. No occupational exposure to chemicals was documented for the third case and he never took alcoholic beverages.

The case with soft-tissue sarcoma diagnosed in 1977 was occupationally exposed to phenoxyacetic acids in 1955–1961 as a railway worker. No other occupational chemical exposure was verified, and he never used alcohol.

### *Exposure*

In the assessment of exposure to different agents, the occupations of the cases and controls were checked from 20 years of age to their retirement. For all cases, 4,806 occupation-years were registered as compared to 9,411 occupation-years for the controls; i.e. comparable numbers. For the controls, 244 years were identified in occupations with potential exposure to organic solvents (repairers of cars and machinery, painters and cleaners) as compared to 215 years for the cases (125 years expected). Regarding farming, 545 years were reported for 27 cases *versus* 1,711 years for 75 controls. The slight over-representation of farmer-years among the controls is in agreement with the observation that farmers in Sweden are only rarely alcoholics (for definition c.f. below). When excluding alcoholics, 36.5% of the cases *versus* 39.0% of the controls had at some time worked in farming, i.e. comparable numbers. For other occupations, no major differences were found between cases and controls.

The exposure to various agents for the 83 cases with HCC and 15 cases with CC is presented in Table II. No major differences were seen in exposure to phenoxy acids or chlorophenols in cases or controls. Alcoholics (c.f. below) might not be involved in occupations with potential exposure to these substances due to the method of working and the fact that alcoholism seems to be less common among farmers than in the whole population. A stratification for alcohol consumption was then made and the risk ratio (point estimate) was calculated: for exposure to phenoxy acids and chlorophenols, 1.8 ($CI_{95}=0.9$–$4.0$); for phenoxy acids only, 1.7 ($CI_{95}=0.7$–$4.4$); and for chlorophenols (high grade) only, 2.2 ($CI_{95}=0.7$–$7.3$); i.e. no significant associations were found. Two exposed cases and one exposed control were alcoholics. The exposure to dichloro-diphenyl-trichloro-ethane (DDT), asbestos, and man-made fibers was comparable in cases and controls. The stated exposure to DDT in forestry was more reliable since this type of work (afforesting with seedlings treated with DDT) was more easy to define than the DDT used in farming to control flies in barns, where other chemicals may also have been used. No differences were seen in exposure to chimney-smoke or municipal incinerators. Reported work with motor-saws in forestry, either in cutting timber or clearing hardwoods, was equally frequent among cases and controls. No significant difference in exposure to exhaust from motor vehicles during leisure time was found between cases and controls. Due to the low number of cases with CC, the differences in some exposures between these cases and controls could be explained by random variation and therefore no risk ratios were calculated for these cases separately.

**Table II** Exposure to different agents in the cases with hepatocellular carcinoma (HCC), cholangiocellular carcinoma (CC), and controls

| | *Exposure %* | | | |
|---|---|---|---|---|
| | *Cases* | | | *Controls* |
| *Agents* | *HCC* | *CC* | *Total* | |
| Total material, number of subjects | (83) | (15) | (98) | (200) |
| Asbestos | 8.4[c] | 13.3 | 9.2 | 9.0[c] |
| Asphalt | 6.0 | 20.0 | 8.1 | 1.5 |
| Chlorophenols | | | | |
| – high grade | 4.8[a] | 13.3 | 6.1 | 3.0[c] |
| – low grade | 2.4 | 0 | 2.0 | 2.0 |
| Coffee | | | | |
| ≥8 cups/day | 20.5[a] | 13.3 | 19.4 | 19.2[b] |
| 2–7 cups/day | 69.9 | 86.7 | 73.5 | 75.3 |
| <1 cup/day | 8.4 | 0 | 7.1 | 5.6 |
| DDT | | | | |
| – farming | 4.8 | 0 | 4.1 | 10.0 |
| – forestry | 6.0[b] | 0 | 5.1 | 4.0[d] |
| Exhaust (leisure time) | 4.8 | 0 | 4.1 | 2.5 |
| Glass fibers | 13.3 | 6.7 | 12.2 | 11.5 |
| Motor saws | | | | |
| – cutting timber | 15.7 | 0 | 13.3 | 16.5 |
| – clearing hardwoods | 7.2[a] | 0 | 6.1 | 7.5[b] |
| Organic solvents | | | | |
| – high grade | 24.1 | 13.3 | 22.4 | 13.5 |
| – low grade | 3.6 | 20.0 | 6.1 | 3.0 |
| Phenoxy acids | 9.6[e] | 0 | 8.2 | 6.5[b, e] |
| Phenoxy acids and chlorophenols (high grade) | 14.5 | 13.3 | 14.3 | 9.5 |
| Smoke (chimney) | 6.0 | 6.7 | 6.1 | 4.0 |
| Smoker (including ex-smokers) | 73.5 | 93.3 | 76.5 | 66.0 |
| Snuff | 30.1[a] | 26.7[a] | 29.6 | 34.0 |
| Tea | | | | |
| ≥8 cups/day | 0[b] | 0[a] | 0 | 0[c] |
| 2–7 cups/day | 9.6 | 20.0 | 11.2 | 16.3 |
| <1 cup/day | 88.0 | 73.3 | 85.7 | 83.7 |

[a]1 subject did not know about exposure.
[b]2 subjects did not know about exposure.
[c]4 subjects did not know about exposure.
[d]6 subjects did not know about exposure.
[e]2 subjects also exposed to chlorophenols, high grade.

*Organic solvents*

Exposure to organic solvents (high grade) was stated by 22.4% of the cases with PLC and 13.5% of the controls. Most cases and controls were exposed to various types of organic solvents such as thinners, turpentine and white spirit. Exposure to trichloroethylene was reported by 2 cases and 1 control. One case had been in contact with perchloroethylene in his work as a drycleaner, but no control had. A risk ratio (point estimate) of 1.8 ($\chi^2=3.8$; $CI_{95}=0.99$–$3.4$) was obtained. For cases with HCC, the risk ratio was 2.1 ($\chi^2=5.1$; $CI_{95}=1.1$–$4.0$) (Tables III, IV). If the subjects who had worked with asphalt were included as potentially exposed to organic solvents, the risk ratio for cases with HCC was 2.4 ($\chi^2=7.9$; $CI_{95}=1.3$–$4.4$). Regarding low-grade exposure to organic solvents, no major differences were found between cases and controls. Due to the low number of exposed individuals, the differences could be explained by random variation.

*Alcohol*

Daily intake of wine was reported by 2 cases and no control. One of the cases was a heavy consumer of spirits (category I; c.f. below) and the other used spirits corresponding to the intermediate group

**Table III** High-grade exposure to organic solvents in cases with primary liver cancer (PLC) and controls

| *Age* | *Cases/Controls* | *Exposed* | *Unexposed* | *Total* |
|---|---|---|---|---|
| 30–55 | Cases | 2 | 5 | 7 |
| | Controls | 2 | 13 | 15 |
| 56–65 | Cases | 3 | 13 | 16 |
| | Controls | 9 | 23 | 32 |
| 66–75 | Cases | 11 | 37 | 48 |
| | Controls | 11 | 85 | 96 |
| 76–80 | Cases | 6 | 21 | 27 |
| | Controls | 5 | 52 | 57 |
| Total | Cases | 22 | 76 | 98 |
| | Controls | 27 | 173 | 200 |
| Crude ratio ratio | | 1.9 | (1.0) | |
| $\chi^2(1)$(Mantel-Haenszel) | | 3.8 | | |
| Rate ratio (Mantel-Haenszel) | | | | |
| – point estimate | | 1.8 | | |
| – $CI_{95}$ | | 0.99–3.4 | | |

**Table IV** High-grade exposure to organic solvents in cases with hepatocellular carcinoma (HCC) and controls

| *Age* | *Cases/Controls* | *Exposed* | *Unexposed* | *Total* |
|---|---|---|---|---|
| 30–55 | Cases | 2 | 3 | 5 |
| | Controls | 2 | 13 | 15 |
| 56–65 | Cases | 2 | 9 | 11 |
| | Controls | 9 | 23 | 32 |
| 66–75 | Cases | 10 | 32 | 42 |
| | Controls | 11 | 85 | 96 |
| 76–80 | Cases | 6 | 19 | 25 |
| | Controls | 5 | 52 | 57 |
| Total | Cases | 20 | 63 | 83 |
| | Controls | 27 | 173 | 200 |
| Crude ratio ratio | | 2.0 | (1.0) | |
| $\chi^2(1)$(Mantel-Haenszel) | | 5.1 | | |
| Rate ratio (Mantel-Haenszel) | | | | |
| – point estimate | | 2.1 | | |
| – $CI_{95}$ | | 1.1–4.0 | | |

(category II). Of the 6 cases and 6 controls drinking wine some times a week, 5 and 4 respectively also consumed spirits every week. Most cases and controls seldom used wine. No association between the use of wine and PLC could thus be demonstrated.

Regarding beer, 4 cases and 1 control consumed more than 3 pints daily. All of them belonged to the group with the highest spirit consumption. Eleven cases and 16 controls who consumed 1 pint of beer daily had an intake of spirits according to category III (crude rate ratio = 3.0; teetotallers as unexposed).

Use of spirits corresponding to more than 370 ml (1 bottle) per week (category I) was reported by 27/83 (32.5%) cases with HCC and 36/200 (18%) controls. These subjects were classified as alcoholics. The calculated risk ratio (point estimated was 4.2 ($\chi^2 = 10.0$; $CI_{95} = 1.8$–10.8) relative to teetotallers (Table V).

**Table V** Use of alcohol in cases with hepatocellular carcinoma (HCC) and controls. (For categories, see test)

| *Age* | *Cases/Controls* | *Exposure categories* *I* | *II* | *III* | *Unexposed* |
|---|---|---|---|---|---|
| 30–55 | Cases | 1 | 1 | 2 | 1 |
| | Controls[a] | 5 | 2 | 6 | 1 |
| 56–65 | Cases | 5 | 2 | 4 | 0 |
| | Controls | 9 | 6 | 13 | 4 |
| 66–75 | Cases | 16 | 6 | 17 | 3 |
| | Controls | 14 | 9 | 52 | 21 |
| 76–80 | Cases | 5 | 3 | 14 | 3 |
| | Controls | 8 | 7 | 29 | 13 |
| Total | Cases | 27 | 12 | 37 | 7 |
| | Controls | 36 | 24 | 100 | 39 |
| Crude rate ratio | | 4.2 | 2.8 | 2.1 | (1.0) |
| $\chi^2(1)$ (Mantel-Haenszel) | | 10.0 | 3.8 | 1.5 | |
| Rate ratio (Mantel-Haenszel) | | | | | |
| – point estimate | | 4.3 | 2.9 | 2.1 | |
| – $CI_{95}$ | | 1.8–10.8 | 0.99–8.7 | 0.9–5.1 | |

[a]Information was not obtained for one control with a psychiatric disease.

A high intake of spirits; i.e. more than 370 ml (1 bottle) per month but less than 1 bottle weekly, gave a risk ratio of 2.9 ($\chi^2 = 3.8$; $CI_{95} = 0.99$–8.7) for HCC (category II). Less consumption corresponding to a maximum of 4 bottles at 370 ml per year gave a risk ratio of 2.1 which was not significant ($\chi^2 = 1.5$; $CI_{95} = 0.9$–5.1). The corresponding risk ratios calculated for the whole PLC group were somewhat lower.

### *Tobacco*

Of the cases with PLC, 76.5% (HCC = 73.5% and CC = 93.3%) reported smoking. Since smoking is related to alcohol intake, a stratification according to consumption of spirits was made (categories I + II, category III + teetotallers). In the first group

(category I and II), 70.2% of HCC, 100% of CC and 76.7% of the controls were smokers. In the second group, the corresponding percentages were 64.3%, 80.0% and 61.4%. The figures also included ex-smokers. No difference was found between cases and controls regarding tobacco snuffing (Table II).

### *Food habits*

Information about food habits was also obtained from the questionnaire. The questions were answered by 75% of the cases and controls and were not supplemented over the phone if unclear. The cases had a slightly higher weekly intake of fried and grilled meat and a slightly lower intake of fish and vegetables than the controls (Table VI). No difference was seen for various other types of food.

**Table VI** Different types of food eaten daily or some times weekly in cases with primary liver cancer and controls

| | *Cases* % | *Controls* % |
|---|---|---|
| Fish | | |
| - cooked | 44.5 (HCC = 42.9) | 71.3 |
| - fried, grilled | 37.0 (HCC = 36.1) | 48.1 |
| - smoked | 2.8 (HCC = 1.6) | 5.6 |
| Meat | | |
| - cooked | 48.7 (HCC = 53.2) | 75.5 |
| - fried, grilled | 68.0 (HCC = 69.9) | 62.5 |
| Pork | 57.5 | 57.9 |
| Sausage | 68.4 | 69.1 |
| Vegetables | | |
| - carrots | 42.5 | 51.0 |
| - salad, tomato, cucumber | 48.0 | 54.9 |
| - other | 29.8 | 37.3 |

### *Previous diseases*

Previous diseases as reported by cases and controls are listed in Table VII. Cirrhosis was reported by 12.2% of the cases with PLC *versus* 1.5% of the controls. Hypertension, neurologic diseases, and hyperlipidaemia were more common in controls than in cases. This might be explained by the median age at death for the controls (67 years), which gave an overfrequency of these diseases when deaths by suicide and cancer were excluded. Gallstones were less frequently reported among cases than controls, which is difficult to explain. Tuberculosis was more common among cases of PLC (7.1%) than among controls (4.0%).

**Table VII** Percentage of previous diseases in cases with hepatocellular carcinoma (HCC), cholangiocellular carcinoma (CC), and controls

| *Diseases* | *Cases* | | | |
|---|---|---|---|---|
| | *HCC* % | *CC* % | *Total* % | *Controls* % |
| Total material, number | (83) | (15) | (98) | (200) |
| Cirrhosis | 13.3 | 6.7 | 12.2 | 1.5 |
| Diabetes | 21.7 | 26.7 | 22.4 | 17.5 |
| Gallstone | 6.0 | 6.7[a] | 6.1 | 16.0 |
| Hepatitis | 2.4 | 0 | 2.0 | 1.0 |
| Hyperlipidaemia | 1.2 | 0 | 1.0 | 6.0 |
| Hypertonia | 21.7 | 26.7 | 22.4 | 17.5 |
| Laesio vascularis cerebri | 10.8 | 6.7 | 10.2 | 11.5[b] |
| Neurological disease | 1.2 | 0 | 1.0 | 3.0 |
| Pancreatitis | 1.2 | 0 | 1.0 | 0[a] |
| Porphyria acuta intermittens (PAI) | 3.6 | 0 | 3.1 | 0 |
| Porphyria cutanea tarda (PCT) | 7.2 | 0 | 6.1 | 0 |
| Tuberculosis | 7.2[a] | 6.1 | 7.1 | 4.0[a] |

[a]1 subject did not know.
[b]3 subjects did not know.

Of HCC, 7.2% had porphyria cutanea tarda (PCT) *versus* none of the CC cases or the controls. Of these 6 cases, 4 were alcoholics and one had haemochromatosis. Since different types of porphyria could not be distinguished in the questionnaires, all the records of the cases and controls were reviewed in this respect also. Porphyria acuta intermittens (PAI) was reported by 3 cases with HCC but by no case with CC and by no control. All these 3 cases had well-differentiated HCC. Cirrhosis could not be evaluated from the preserved pathological specimens.

### *Drugs*

An intake of clofibrate was not reported by any case but by 5 controls, which was related to the fact that hyperlipidaemia was more common among the controls (Table VIII). The use of antihypertensive drugs was also more common in the controls. Among the cases, 6.1% had been treated with tuberculostatic drugs compared to 2.5% among the controls.

### Discussion

PLC is one cancer with the highest registered increase in the incidence rate in Sweden and this can hardly be explained by purely diagnostic factors. Sweden is, however, still a low-incidence

**Table VIII** Intake of drugs in cases with primary liver cancer (PLC) and controls (percentage)

| | *Cases* % | *Controls* % |
|---|---|---|
| Total material, number | (98) | (200) |
| Hyperlipidaema | | |
| – clofibrate | 0 | 3.0 |
| Hypertonia | | |
| – hydralazine | 1.0 | 6.0 |
| – methyldopamine | 3.9 | 9.5 |
| Tuberculosis | | |
| – chemotherapy | 6.1 | 2.5 |

region with an age-specific incidence typical for such regions. In countries with high incidence, the peak incidence shifts to younger age groups. In Mozambique, the incidence in males under 40 years of age is 500 times greater than in US white men of the same age, whereas after 65 years of age the excess is only two-fold, which might indicate a different aetiology in these age groups (Nagasue, 1982).

Esposure to polyvinyl chloride can induce haemangiosarcoma in the liver (Creech & Johnson, 1974). One of our 3 cases with haemangiosarcoma had such exposure. The case with unspecified soft-tissue sarcoma in the liver was exposed to phenoxy acids in 1955–1961 with a latency period of 22 years. Previous studies have linked exposure to phenoxy acids with an increased risk of soft-tissue sarcoma (Hardell & Sandström, 1979; Eriksson *et al.*, 1981).

In several studies, primary liver cancer of the HCC type has been related to persistent hepatitis B viral (HBV) infection (HBsAg carriers). The relative risk has been estimated to be 10–70 among carriers in different populations (Trichopoulos *et al.*, 1978; Tabor *et al.*, 1977). Since the prevalence of HBsAg positive carriers in Sweden is less than 1%, the aetiological fraction can be estimated to be 10% or less (Trichopoulos, 1981). Our cases were diagnosed between 1974 and June 1981 at different hospitals in our admission region. No serological data regarding HBV infection were available on our cases. Persistent HBV infection is more frequent in alcoholics than in the general population (Brechot *et al.*, 1982). An aetiological interaction between HBV and alcohol may be important and should be studied more. Aflatoxin has been shown to be a liver carcinogen in countries with high intake (Linsell & Peers, 1977). Sweden belongs to an area with a low intake of aflatoxins and it seems unlikely that aflatoxins contribute to PLC to any appreciable extent.

In our study, alcohol seemed to be the most important factor in inducing PLC. Information about alcohol consumption was obtained in the questionnaires. To check the accuracy of the answers, records of the cases and controls from hospitals and health centers were scrutinized regarding statements about alcohol habits. For 20 cases and 12 controls classified as alcoholics (spirits = category I; see above) according to the information in the questionnaires, this was in agreement with the records. Thirteen cases and 24 controls were classified as alcoholics but no data about alcohol consumption were found in their records. Only one case and one control were classified as alcoholics according to the records, but not according to the questionnaires from which they were classified as caregory III consumers. The questionnaire thus seemed to give fairly reliable data concerning the use of alcohol.

Persons with high intake of wine and beer usually also had a high intake of spirits which is why these factors were difficult to study separately. No association between PLC and the intake of wine or beer *per se* was found in this study. The aetiological fraction of HCC which could be attributed to alcohol was 25%, if only alcoholism was considered, and 34% if regular intake was also included. Usually, alcohol causes cirrhosis of a micronodular type, but a macronodular pattern can be seen in reformed alcoholics. Cirrhosis, mostly of the macronodular type, is seen in 60–90% of HCC (Linsell & Higginson, 1976). If alcohol can induce HCC more directly or if tumour development always requires existent cirrhosis is not known. HCC, however, has also been reported in alcoholics without cirrhosis (Anthony, 1976). Of our cases, 34% were primarily reported to have cirrhosis. During histopathological re-examination, the material was found to be inadequate for an evaluation of cirrhosis since in most cases only specimens from the cancer and not from the surrounding liver tissue were available. Regarding social drinkers, a crude risk ratio of 2.1 was found, which was insignificant. CC of the intrahepatic type is not considered to be associated with alcoholism or cirrhosis (Okuda, 1977). Of the 15 cases with CC, 6 were alcoholics with a crude ratio of 3.3, which was not significant, ($\chi^2 = 2.1$; $CI_{95} = 0.7–16.1$). Due to the low number of cases with CC in our study, no conclusions could be drawn.

Smoking was reported somewhat more frequently by the cases than by the controls. After stratification for alcohol, no increased risk by smoking could be found. Some other studies have indicated an association between smoking and PLC which could not be reproduced in our study (Lam *et al.*, 1982; Trichopoulos *et al.*, 1980).

In the assessment of exposure to different agents, both a history of previous occupations and exposure to individual chemical substances were obtained from the questionnaires. Potential confounding problems relating to sex, age, place of residence, and year of death were avoided by matching the controls to the cases. To avoid bias introduced by the interviewer, information was supplemented over the phone without knowing if the subject was the next-of-kin of a case or control. Since all the cases were dead, deceased controls were selected in order to avoid differences in recall between live subjects and deceased subjects' next-of-kin.

No major differences in exposure to different agents was found between cases and controls except for organic solvents and asphalt. An elevated risk of PLC in occupations with exposure to organic solvents has recently been reported (Stemhagen *et al.*, 1983). In our study, the relative number of person-years in occupations with potential exposure to organic solvents was higher in cases than in controls. An exposure to organic solvents gave an approximately 2-fold increase in the risk of HCC. Asphalt workers were also over-represented among the cases. No increased risk of HCC among asphalt workers has been described previously. Asphalt workers are potentially exposed to organic solvents, and asphalt also contains different polyaromatic hydrocarbons with carcinogenic properties. In animal studies, dioxins have induced HCC among other cancer types (van Miller *et al.*, 1977; Kociba *et al.*, 1978). They are found as impurities in phenoxy acids and chlorophenols. No association between exposure to these chemicals and PLC was found in this investigation, although a formal risk of 1.8 was obtained for combined exposure.

The intake of tuberculostatic drugs was more frequent in the cases than the controls, with a crude rate ratio of 2.5. The most common combination was isoniazid, streptomycin and p-aminosalicyclic acid. Isoniazid is of interest, since it is metabolized to an alkylating agent in the liver (Sherlock, 1979).

Porphyria cutanea tarda (PCT) is usually associated with an underlying liver disease (Doss *et al.*, 1976). Of our 6 cases with PLC and PCT, 4 were alcoholics and 1 had haemochromatosis. In a series of 10 cases with PCT who developed PLC, all were alcoholics with liver cirrhosis (Solis *et al.*, 1982).

Porphyria acuta intermittens (PAI) is an "inborn error of metabolism" inherited as a dominant trait. It is characterized by a reduced urosynthetase activity in the synthesis of heme. The disease is diagnosed by increased excretion of two precursors (aminolevulinic acid and porphobilinogen (PBG)) in the patient's urine and/or decreased urosynthetase activity in erythrocytes. Due to the familial occurrence, the prevalence of PAI has been reported to be higher in northern Sweden than in the rest of the country; i.e. 1 case per 1000 inhabitants (Waldenström, 1969). Several of the cases with PAI have been traced to one person in the 17th century living in northern Sweden.

The three cases with PAI and HCC involved in this investigation were 69, 66 and 65 years old at diagnosis. One of them consumed alcohol, which provoked acute attacks of PAI. Since the prevalence of PAI in the population in our area is expected to be 1 case in 1000 persons, the finding of 3 cases with PAI among our 83 cases with HCC could not be explained by chance. As mentioned in the introduction, an association between HCC and PAI had previously been observed in northern Sweden (Lithner & Wetterberg, 1984).

In conclusion, this investigation indicates an association between alcohol abuse and hepatocellular carcinoma, while no conclusions could be drawn regarding cholangiocellular carcinoma. An increased risk was also found for occupational exposure to organic solvents. The inherited disease porphyria acuta intermittens certainly constituents a risk factor, but further studies are warranted for a more quantitative risk estimation. Asphalt work and the intake of tuberculostatic drugs may be other risk factors. Hepatitis B virus infection and cirrhosis, which are factors of great aetiological interest could not be evaluated in the present study due to the lack of sufficient data.

This study was supported by grants from the Swedish Cancer Society (project number 1046-B83-08XC).

### References

ANTHONY, P.P. (1976). The background of liver cell cancer. In: *Liver Cell Cancer*. (Eds. Cameron *et al.*), Oxford: Elsevier Sci. Publ. Co., p. 93.

BRECHOT, C., NALPAS, B., COUROUCE, A.M. & 5 others. (1982). Evidence that hepatitis B virus has a role in liver-cell carcinoma in alcoholic liver disease. *N. Engl. J. Med.*, **306**, 1384.

CADY, B. (1983). Natural history of primary and secondary tumors of the liver. In: *Semin. Oncol.*, June 1983, p. 127.

CREECH, L.J. Jr. & JOHNSON, M.N. (1974). Angiosarcoma of liver in the manufacture of polyvinyl chloride. *J. Occup. Med.*, **16**, 150.

DOSS, M., SCHERMULY, E., LOOK, E. & HENNING, H. (1976). Enzymatic defects in chronic hepatic porphyrias. In: *Porphyrins in Human Diseases*. (Ed. Doss), Basel: Karger, p. 286.

ERIKSSON, M., HARDELL, L., BERG, T., MÖLLER, T. & AXELSON, O. (1981). Soft-tissue sarcomas and exposure to chemical substances: A case-referent study. *Br. J. Ind. Med.*, **38**, 27.

HARDELL, L. (1981). Relation of soft-tissue sarcoma, malignant lymphoma and colon cancer to phenoxy acids, chlorophenols and other agents. *Scand. J. Work Environ. Health*, **7**, 119.

HARDELL, L. & SANDSTRÖM, A. (1979). Case-control study: Soft-tissue sarcomas and exposure to phenoxyacetic acids or chlorophenols. *Br. J. Cancer*, **39**, 711.

HUEPER, W.C. & CONWAY, W.D. (1964). *Chemical Carcinogenesis and Cancers*, Springfield, Illinois: Thomas, p. 40.

KOCIBA, R.J., KEYES, D.G., BEYER, J.E. & 9 others. (1978). Results of a two-year chronic toxicity and oncogenicity study of 2,3,7,8-tetrachloro-dibenzo-p-dioxin in rats. *Toxicol. Appl. Pharmacol.*, **46**, 279.

LAM, K.C., MIMI, C., LEUNG, J.W.C. & HENDERSON, B.E. (1982). Hepatitis B virus and cigarette smoking: Risk factors for hepatocellular carcinoma in Hong Kong. *Cancer Res.*, **42**, 5246.

LINSELL, C.A. & HIGGINSON, J. (1976). The geographic pathology of liver cell cancer. In: *Liver Cell Cancer*. (Eds. Cameron *et al.*), Oxford: Elsevier Sci. Publ. Co., p. 1.

LINSELL, C.A. & PEERS, F.G. (1977). Aflatoxin and liver cell cancer. *Trans. R. Soc. Trop. Med. Hyg.*, **71**, 471.

LITHNER, F. & WETTERBERG, L. (1984). Hepatocellular carcinoma in patients with acute intermittent porphyria. *Acta Med. Scand.*, (In Press).

MANTEL, N. & HAENSZEL, W. (1959). Statistical aspects of the analysis of data from retrospective studies of disease. *J. Natl Cancer Inst.*, **32**, 719.

MIETTINEN, O.S. (1972*a*). Components of the crude risk ratio. *Am. J. Epidemiol.*, **96**, 168.

MIETTINEN, O.S. (1972*b*). Standardization of risk ratios. *Am. J. Epidemiol.*, **96**, 383.

MIETTINEN, O.S. (1976). Estimability and estimation in case-referent studies. *Am. J. Epidemiol.*, **103**, 226.

NAGASUE, N. (1982). The epidemiology and etiology of prma liver cancer: A current trend. *Fukuoko Acta Medica*, **73**, 281.

NATIONAL BOARD OF HEALTH AND WELFARE. (1983). The Cancer Registry. *Cancer Incidence in Sweden 1980.*

NORKRANS, G., LINDBERG, J., WAHL, M., HERMODSSON, S. & LINDHOLM, A. (1983). Exposure to hepatitis B among hospital employees, policemen and healthy blood donors in Gothenberg. *Läkartidningen 80*, **36**, 3176.

OKUDA, K., KUBO, Y., OKAZAKI, N. & 10 others. (1977). Clinical aspects of intrahepatic bile duct carcinoma including bilar carcinoma: A study of 57 autopsyproven cases. *Cancer*, **39**, 232.

PERSHAGEN, G. & AXELSON, O. (1982). A validation of questionnaire information on occupational exposure and smoking. *Scand. J. Work, Environ. Health*, **8**, 24.

POPPER, H. (1979). Hepatic cancers in man: Quantitative perspectives. *Environ. Res.*, **19**, 482.

SHERLOCK, S. (1979). Hepatic reaction to drugs. *Gut*, **20**, 634.

SOLIS, J.A., BETANCOR, P., CAMPOS, R. & 4 others. (1982). Association of porphyria cutanea tarda and primary liver cancer. *J. Dermatol.*, **9**, 131.

STEMHAGEN, A., SLADE, J., ALTMAN, R. & BILL, J. (1983). Occupational risk factors and liver cancer: A retrospective case-control study of primary liver cancer in New Jersey. *Am. J. Epidemiol.*, **117**, 443.

TABOR, E., GERETY, R.J., VOEGEL, C.L. & 4 others. (1977). Hepatitis B virus infection and primary hepatocellular carcinoma. *J. Natl Cancer Inst.*, **58**, 1197.

TRICHOPOULOS, D. (1981). The causes of primary hepatocellular carcinoma in Greece. *Prof. Med. Virol.*, **27**, 14.

TRICHOPOULOS, D., TABOR, E., GERETY, R.J. & 4 others. (1978). Hepatitis B and primary hepatocellular carcinoma in a European population. *Lancet*, **ii**, 1217.

TRICHOPOULOS, D., MACMAHON, B., SPARROS, L. & MERIKAS, G. (1980). Smoking and hepatitis B-negative primary hepatocellular carcinoma. *J. Natl Cancer Inst.*, **65**, 111.

VAN MILLER, J.P., LALICH, J.J. & ALLEN, J.R. (1977). Increased incidence of neoplasms in rats exposed to low levels of 2,3,7,8-tetrachlorodibenzo-p-dioxin. *Chemosphere*, **6**, 537.

WALDENSTRÖM, J. (1969). Porphyria. In: *Medicinskt Kompendium, II.* (Eds. Iversen *et al.*), Köpenhavn: Nyt Nordisk Forlag. Arnold Buschk., p. 1132.